# University of Buffalo—Commencement Week, 1906

**Published:** 1906-07

**Authors:** 


A Monthly Review of Medicine and Surgery.

*EDITOR:*

WILLIAM WARREN POTTER, M. D.

All communications, whether of a literary or business nature, books for review and exchanges, should be addressed to the editor 284 Franklin St., Buffalo, N. Y.

Vol. Lxi. JULY, 1906. No. 12

### University of Buffalo—Commencement Week, 1906.

THE exercises relating to the sixtieth annual commencement of the department of medicine, the nineteenth annual commencement of the department of pharmacy, the eighteenth annual commencement of the department of law, and the fourteenth annual commencement of the department of dentistry were held at the Teck theater Friday morning, June 1, at 11 o'clock. Degrees were conferred upon 44 candidates in medicine, 59 in pharmacy, 20 in law, and 34 in dentistry, all of whom had been previously recommended to the council by the several faculties. The vice-chancellor, Charles P. Norton, Esq., conferred the degrees upon the candidates in each of the four departments, observing the usual legal forms in vogue in the university since its establishment in 1846.

The names of those who received degrees in the department of medicine are as follows:

Harmon Hadley Ashley, George W. Bachmann, Charles Bathaglia, Jacob William Bayliss, Vincent B. Beseynski, William Henry Billings, Jr., Robert Burtis Blanchard, Harvey W. Bodamer, Frederick Berkley Bond, William Warren Britt, Israel Cohn, Harley U. Cramer, William S. Driscoll, Otto Robert Eichel, Sara Elizabeth Green, Arthur R. Gibson, Clara Olive Griffin, George M. Growney, John Joseph Hanavan, Edith Rebecca Hatch, Albert Andrew Herschler, John Van Ord Hibbard, John Conrad Hoeffler, Moses Holtz, Edward Enos Hopkins, Luther Marshall Jayne, Joseph N. Kiefer, Ray W. Kimball, Jesse Goldberg Levy, Verne Artemas Mann, Joseph C. O'Gorman, Winfield Augustine Peterson, Ralph Sumner Pettibone, Merle Albert Place, Gardner Ellis Robertson, Albert Michael Rooker, William Joseph Ryan, Elihu Standish, Arthur Porter Squire, Luther Allen Thomas, Patrick Henry Whalen, Russell H. Wilcox, Cynthia Evelyn Williamee, Charles Arthur Wisch.

The total number of graduates was 157,—44 of whom received the degree of doctor of medicine, 59 the degree of bachelor in pharmacy, 20 the degree of bachelor of laws, and 34 the degree of doctor of dental surgery.

The honor list in medicine with the standing of each is as follows:

John Van Ord Hibbard, 94.92; Edith Rebecca Hatch, 94.85; Albert Michael Rooker, 94.07; Cynthia Evelyn Williamee, 92.67; George W. Bachmann, 90.46.

The hippocratic oath was administered to the candidates of the class in medicine by Dr. Matthew D. Mann, dean of the faculty. The candidates were presented to the vice-chancellor by the secretaries of the respective faculties, Dr. Eli H. Long serving in that capacity for the faculty of medicine.

After the announcement of honors, Dr. Edward N. Brush, of Baltimore, superintendent of the Sheppard and Enoch Pratt Hospital, a graduate of the University of Buffalo, class of 1874, delivered the annual commencement address to the combined classes. This masterful paper will be published in full text in a future number of the JOURNAL. The invocation was made by the Rev. George B. Richards and the benediction was pronounced by the Right Reverend Charles H. Colton, D. D., Bishop of Buffalo. The exercises were interspersed with appropriate music, but the usual gifts of flowers were omitted.

At the conclusion of the graduating ceremonies the faculty in medicine entertained the medical graduates and visiting alumni at luncheon, which was served at the University club. At the conclusion of the luncheon a pleasant surprise came to all. Dr. William Henry Billings, Jr., on behalf of his associates of the just-now graduated class, presented a purse of $500 to the university extension fund. Vice-chancellor Charles P. Norton accepted the gift in appropriate terms, congratulating the class on this generous exhibition of loyalty to its Alma Mater.

ALUMNI ASSOCIATION.

The Alumni association of the medical department held its thirty-first annual meeting Tuesday, Wednesday Thursday and Friday, May 29, 30, 31, and June 1, 1906. The days were given over to clinics at the several hospitals as announced in the JOURNAL for June, luncheons were served each noon at the University club, while the evenings were devoted to exercises at Alumni hall and smokers in the college library.

The annual business meeting was held at Alumni hall Tuesday evening at 8 o'clock, at which time the president, Dr. Fridolin Thoma, '87, of Buffalo, delivered his address, officers for the ensuing year were elected and other routine business was transacted. Dr. Grover W. Wende at 9 o'clock exhibited curiosities and anomalies in dermatology with the aid of the lantern. The smoker began at 10 o'clock in the college library which included a collation and music by the university mandolin and glee clubs.

The clinics were continued during Wednesday, May 30, the teaching staff gave a luncheon to the Alumni at the University club at noon, at 5 p. m. Dr. Willis G. Gregory at Alumni hall exhibited therapeutic curiosities with the lantern, at 8 p. m. at the college building a reunion was held of the classes of '56, '66, '76, '86, and '96, at 9 p. m. Dr. H. G. Matzinger discoursed on psychopathy and Dr. H. U. Williams on pathology, and at 10 p. m. the usual smoker closed the entertainment for the day. The university mandolin and glee clubs again furnished delightful music for the assemblage.

The clinics were further continued on Thursday, a luncheon at noon was served at the University club under the auspices of Doctors Lucien Howe, William C. Phelps, Ernest Wende, Peter W. Van Peyma, James W. Putnam, Floyd S. Crego, Elmer G. Starr, Henry C. Buswell, DeWitt H. Sherman, and Fridolin Thoma, and in the evening at 8 o'clock Dr. Denslow Lewis, of Chicago, delivered an address on the "Social Evil," at Alumni hall. The audience which listened to Dr. Lewis was large and enthusiastic and the discussion which was participated in by the Rev. Frank S. Fitch, Judge George A. Lewis, Dr. M. D. Mann, Dr. W. W. Potter and others, was spirited. The usual smoker and collation with music brought the meeting of the Alumni association for 1906 to an end.

---

The following-named officers of the alumni association of the Medical department were elected for the current year: president, William R. Campbell, Niagara Falls; vice-presidents, A. A. Jones, Buffalo; E. C. Scofield, Bemus Point; G. A. Himmelsbach, Buffalo; C. H. Preisch, Lockport; Gertrude Bebee, Buffalo; treasurer, H. K. DeGroat, Buffalo; and secretary, N. G. Russell, Buffalo. The place of the late D. W. Harrington on the board of trustees was taken by Fridolin Thoma. The others are Walden M. Ward, North Collins; A. W. Henckell, Rochester; A. W. Bayliss, Buffalo, and Robert P. Bush, Horseheads, all re-elected.

---

Decennial Class reunions ending in "6"—namely, '56, '66, '76, '86, and '96,—were held Wednesday evening, May 30. Only one man was on hand from the class of '56, George W. Barr, of Titusville, Pa. Drs. William C. Phelps, Conrad Diehl and P. W. Van Peyma of Buffalo, and Dr. Dubois, of Camden, N. Y., represented the class of '66. Of the 35 members of the class of '76 only 10 were present and they elected officers as follows: Dr. B. H. Putnam of North East, Pa., president; Dr. F. L. June of Waterport, secretary and treasurer; vice-presidents, Dr. Walter D. Greene, Dr. A. A. Hubbell, Dr. John G. Miller of Lancaster, Dr. William J. Faulkner of Youngstown, N. Y., and Dr. John G. Van Pelt of Niagara Falls. Dr. A. L. Richman of Morton and Dr. Charles P. Eller of Buffalo were on hand from the class of '86. From the class of '96 a large number were present.

---

The problems that confront the executive, health, and police departments of Buffalo, and which await reasonably prompt solution are many. Among the most important may be mentioned unnecessary noises, soft coal smoke, spitting on the sidewalks, and filthy abbattoirs. These are all intimately related to the health of our citizens and should be dealt with from that viewpoint.

